# Network theory may explain the vulnerability of medieval human settlements to the Black Death pandemic

**DOI:** 10.1038/srep43467

**Published:** 2017-03-06

**Authors:** José M. Gómez, Miguel Verdú

**Affiliations:** 1 Dpto de Ecología Funcional y Evolutiva, Estación Experimental de Zonas Aridas (EEZA-CSIC), Ctra. de Sacramento s/n, La Cañada de San Urbano, 04120, Almería, Spain; 2 Dpto de Ecología, University of Granada, Granada, Spain; 3 Centro de Investigaciones sobre Desertificación (CSIC-UV-GV), Valencia, Spain

## Abstract

Epidemics can spread across large regions becoming pandemics by flowing along transportation and social networks. Two network attributes, transitivity (when a node is connected to two other nodes that are also directly connected between them) and centrality (the number and intensity of connections with the other nodes in the network), are widely associated with the dynamics of transmission of pathogens. Here we investigate how network centrality and transitivity influence vulnerability to diseases of human populations by examining one of the most devastating pandemic in human history, the fourteenth century plague pandemic called Black Death. We found that, after controlling for the city spatial location and the disease arrival time, cities with higher values of both centrality and transitivity were more severely affected by the plague. A simulation study indicates that this association was due to central cities with high transitivity undergo more exogenous re-infections. Our study provides an easy method to identify hotspots in epidemic networks. Focusing our effort in those vulnerable nodes may save time and resources by improving our ability of controlling deadly epidemics.

Most important pandemics in human history, such as cholera, plague, influenza, HIV/AIDS, malaria, or severe acute respiratory syndrome (SARS), have propagated across large territories following transportation, commercial and travelling networks^1^,^2^,^3^. Pathogen invasion of distant areas through air transportation networks is promoting the appearance of emerging infectious diseases in new, formerly disease-free territories, such as malaria in Brazil^4^ and dengue in North America^5^. The transmission dynamics of infectious diseases have been investigated extensively using complex network theory in which nodes represent species, populations, individuals, etc., which are connected through sexual, social, or commercial contacts^3^,^6^.

Two topological and structural properties of networks mediate the spreading dynamics of diseases^3^,^7^,^8^,^9^,^10^,^11^,^12^,^13^,^14^,^15^,^16^,^17^,^18^,^19^,^20^,^21^,^22^: transitivity (the propensity of nodes of clustering together^9^,^11^,^12^) and centrality (the number and intensity of connections with other nodes 3). Diseases spread more efficiently in highly transitive networks^3^,^13^,^14^,^15^. In contrast, factors decreasing transitivity, such as the existence of structural holes that disconnect neighboring nodes, hinders the traffic throughout the network^3^. Nodes displaying high transitivity, because are located in denser regions of the network, can be reached multiple times by disease through different routes of transmission^21^,^22^. The occurrence of reinfections highly increases the virulence of the pathogens and the mortality rate of the hosts^23^,^24^. On the other hand, central nodes are more important sources of diseases than peripheral nodes, both in sexual, contact and ecological networks^3^,^6^,^9^,^16^,^17^,^18^,^19^,^20^. They also become infected earlier, while isolated nodes become infected later or even avoid infection^13^. This suggests that nodes bearing high values of centrality and transitivity in epidemic networks will be more vulnerable and will undergo more mortality than peripheral nodes.

In this study we investigate how network centrality and transitivity influence vulnerability to diseases of human populations by examining one of the most devastating pandemic in human history, the fourteenth century plague pandemic called Black Death. This epidemic ravaged Europe between 1346 and 1353, killing between 30% and 50% of its population^2^. The causative agent and etiology of the medieval Black Death are still debated. Most researchers accept that this infectious disease was caused by the Gammaproteobacteria *Yersinia pestis*. Its transmission to humans is believed to have occurred mostly through the action of some vectors, specifically the Oriental rat fleas (*Xenopsylla cheopsis*) living on the black rats (*Rattus rattus*)^2^,^25^. Humans are infected after being bitten by a flea bearing the diseases. Other researchers have suggested that human contagion is direct through airborne transmission^2^. Irrespective of the specific transmission vector, all researchers agree that the spread of the Black Death was tightly associated to the trade routes^2^. Historical^2^ and epidemiological^26^,^27^ evidence suggest that the Black Death originated in Central Asia and travelled westward along the Silk Road, reaching the city of Caffa in Crimea in 1343. From there, the plague entered into Europe following the main route of an expanding trade network and reaching most human settlements, from large cities and seaports to small villages and hamlets. Old World in the Middle Age held a dense network connecting many localities all over the region. We hypothesize that, as occurring in biological parasite networks, the centrality and local transitivity of the human settlements (cities, hereafter) in the medieval network would be positively associated to their vulnerability to the Black Death. We propose that this relationship is mediated by a higher probability of re-infection of those cities having higher centrality and transitivity. To test this idea, we 1) built the medieval network and empirically explored the relationship between the network attributes of the cities and their mortality due to the Black Death, and 2) simulated how often a city is reached by the disease as a consequence of its position in the medieval network.

## Results

### Description of the medieval network

**Figure 1:**
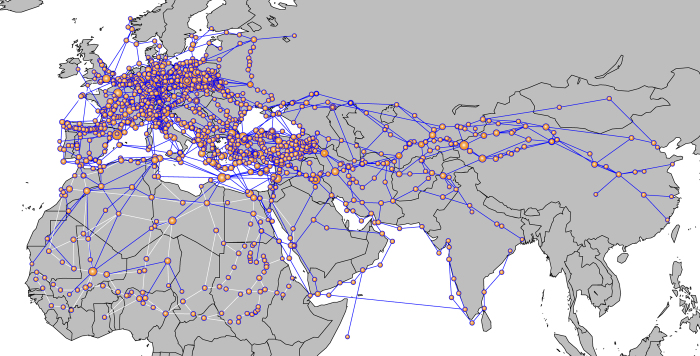
Representation of the network connecting the medieval European and Asian cities through pilgrimage and commercial routes during XIV century. Bubble size is proportional to the centrality value of the cities. Blue links indicate trading routes and white links indicate pilgrimage routes (note that many pilgrimage routes travelled across Europe are hidden by the more numerous trade routes). The spatial network and the map was built using R^37^ (see Supplementary Methods for the scripts).

The studied network included information on 2084 trade and pilgrimage connections between 1311 Old World cities (including North Africa, Europe and Asia), with 1013 cities being connected by trading routes and 403 cities connected by pilgrimage routes, with some cities appearing in both trade and pilgrimage routes (Fig. 1; Supplementary Table S1). Network density, measured as the ratio of the number of connections and the number of possible connections between all cities in the network, was lower than 1% in the three studied networks (Supplementary Table S1). The degree distribution of the network was better fitted to an exponential (R^2^ = 0.991) than to a power-law (R^2^ = 0.88) function (Supplementary Fig. S1).

Network transitivity was around 10% for the overall and the trade networks and only 5% for the pilgrimage network. In all cases, network transitivity was significantly higher than expected in a random network (Supplementary Table S1). The values of local transitivity were very similar to the global values (Overall network = 0.098 ± 0.006; Trade network = 0.063 ± 0.020; Pilgrimage network = 0.018 ± 0.017). However, most cities had transitivity values equal to zero (Supplementary Fig. S2), indicating that they did not form closed triples.

As for centrality, the mean city degree was 3.18 ± 0.06 for the overall network, indicating that as an average a city was connected to other three cities. Nevertheless, degree ranged between 1 and 19, with most cities having a degree between 1 and 5 (Supplementary Fig. S3). Mean degree was 2.49 ± 0.06 for the trade network and 0.69 ± 0.03 for the pilgrimage network. Closeness centrality shared the same pattern; most cities had very low values whereas some cities had high values (Supplementary Figs S3–S5).

There were significant correlations between both centrality metrics in the three types of networks (p < 0.0001 in all cases, N = 1311 cities; Pearson product-moment correlation; Supplementary Table S2), and these correlations were higher than the correlation expected in a network with the same degree distribution but randomly reshuffling the links among the cities (Table S2). Local transitivity was positively correlated with closeness in the overall network (Supplementary Table S3) and negatively correlated with degree (Supplementary Fig. S6).

### Relationship between city mortality and network attributes

We found information about mortality on 58 cities (Supplementary Table S4). The centrality of these cities expanded through the whole range of centralities observed in the whole set of cities (Supplementary Table S4). The average mortality rate of this pool of cities was 51 ± 2% (mean ± 1 SE), ranging between 0% in Turku and over 70% in Ajaccio, Montpellier, Avignon and other southern France cities. There was positive spatial (r = 0.32, P = 0.005, Mantel test) and temporal (r = 0.20, P = 0.019, Mantel test) autocorrelation in mortality rates, indicating that nearby cities or those cities infected at the same time had similar rates of mortality.

**Figure 2:**
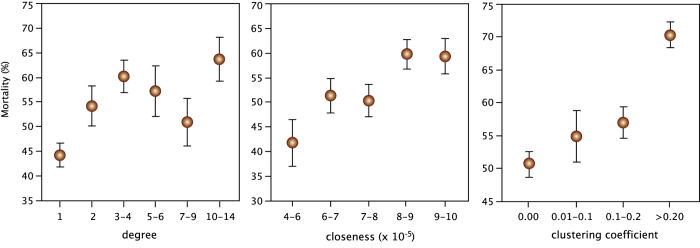
Relationship between mortality and network attributes of the cities. Data were grouped into categories for illustration purposes, but statistical analyses testing the effect of each centrality metric on mortality rates were done following spatially-explicit GLMs using original values for individual cities (Table 1).

There was a significant positive relationship between local transitivity and mortality rate of the cities both in the overall network as well as in the trade network (Table 1, Fig. 2). This means that cities located in dense regions of the network were more affected by the plague than cities located in sparse regions. However, it could not be computed for the pilgrimage network because all cities but two had open triples. Similarly, we found a significant positive relationship between mortality rate and the centrality metrics for the overall network (Table 1, Fig. 2). Central cities underwent higher mortality rate. These relationships maintained significant for degree in the trade network (Table 1).

### Simulating the effect of centrality and transitivity on the probability of multiple infections

**Figure 3:**
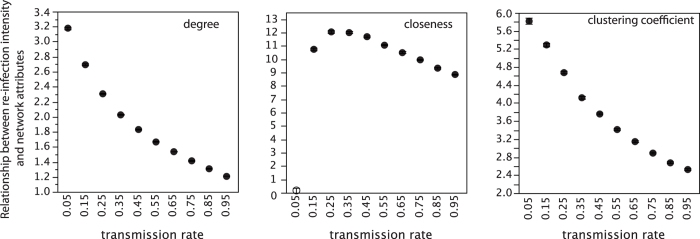
Relationship between the network attributes of the cities and the probability of multiple infections. It is shown the mean spatially-explicit coefficient relating each centrality and transitivity metric and the number of infection undergone by each city in the medieval network under different transmission rates of the plague (N = 1000 simulations per transmission rate and network attribute). In black, significant relationships.

The relationship between number of infections per city and its centrality and transitivity was significantly positive in all cases except in the scenario with lowest infectivity for closeness (Fig. 3). This means that, irrespective of the transmission rate, central and transitive cities tend to be reached by the diseases multiple times, whereas peripheral cities with low transitivity are reached by the disease few times. We found that, although significant, the relationship between multiple infections and centrality and transitivity steadily decreased when the diseases became more infective (Fig. 3). That is, the advantage of being isolated decreased when the disease is severe and transmit very fast.

## Discussion

### Structure of the medieval network

The topology of the medieval network had a transitivity significantly higher than that expected in random networks, a property shared with small-world networks^28^. In particular, the exponential degree distribution of the medieval networks suggests that they were single scale small-world networks. This means that cities were equally connected among neighbour cities and there were not cities with many links. In fact, the highest degree observed was just 19 even although there were 1311 cities. Degree provides a description of network connectivity based on the individual components and assesses the importance of a node based on its reachability. Interestingly, degree was significantly correlated with closeness, a metrics defining the flow pathways along the entire network. Cities with high values of closeness act as bridges, connecting one part of the network to another that would otherwise be sparsely or not connected at all. Consequently, this correlation indicates that the importance of a given city for the disease transmission in the medieval network appears both at local and global scales.

The observed high transitivity in the medieval networks suggests that the dynamics of the infection was predominantly a local process^9^. Under these circumstances, the probability of infection of a given city probably depended more on the transmission from the surrounding cities than from distant cities^9^. Despite of this, infection spreading tends to be fast in networks bearing high transitivity^28^. The correlations between both centrality metrics and between clustering coefficient and closeness insinuate that the infection probably arrived sooner to those cities located in dense and highly interconnected subsets of cities. All these features may explain why the Black Death invaded so easily and fast most places from China to Iberian Peninsula and British Islands in less than a decade^2^.

### Relationship between city centrality, transitivity and mortality rate

We found that nearby cities had similar mortality rates. In the same way, we also found that those cities being infected at the same time had similar mortality rates. Spatio-temporal autocorrelation in mortality rate was found even despite we connected cities through linear routes rather than through the real routes across sea and land. Theoretical studies have shown that the spatial structure of the networks strongly influences the transmission dynamics of infectious disease^9^. In fact, spatio-temporal travelling waves are frequent in epidemics^29^,^30^. The observed autocorrelation in our study system agrees with these theoretical expectations and indicates the occurrence of a spatio-temporal dynamics in the spread of Black Death across Eurasia. Future research should consider more sophisticated modelling to correctly determine the influence of space and time in the dynamics of the Black Death.

Cities exhibiting higher centrality and local transitivity underwent higher rates of mortality than peripheral ones. This pattern has been detected despite we only considered processes related with the transmission of the disease between cities, and omitted the processes occurring within cities and related to the transmission among individuals. In addition, this significant relationship was found after controlling for the spatio-temporal dynamics observed in the spread of the plague in Europe during the fourteenth century, and even despite the uncertainty of data on mortality during the fourteenth century^2^, suggesting thereby that the association was robust. Furthermore, this relationship occurred irrespective of the metric used to calculate centrality, indicating that those cities reached sooner and easier by the diseases are those supporting higher rates of mortality. Nevertheless, the mortality intensity may be reduced in central cities by means of drastic practices. For example, in Milan the authorities struggled against the plague very efficiently^2^. Consequently, despite its high centrality, the city remained functionally isolated and the impact of the disease was very mild.

The relationship between transitivity and mortality was stronger in the overall network than in any of the other two partial networks, an outcome suggesting the occurrence of a synergic effect of the trading and pilgrimage networks. This outcome agrees with some recent studies showing that epidemics spread easier in multiple networks^22^.

### A potential mechanism explained the observed pattern

The pattern we have found in this study may be explained by the occurrence of multiple infections. Recent molecular evidence suggests that multiple infections were frequent during the Black Death pandemic. It has been recently shown that different clones of *Yersinia pestis* were involved in the Black Death pandemic^27^. Similarly, according to molecular analyses in human skeletons, it seems that *Y. pestis* entered Europe several times^26^,^27^. Under this scenario of multiple infections, we presume that central, well-connected cities with high local transitivity had higher likelihood of being invaded multiple times by pathogens. Central cities are those cities that, by having a peculiar history, structure, size, etc., are highly connected with the rest of the cities in the medieval network. This means that those cities were receiving pilgrims, goods and people from many other different cities. This density of commercial and spiritual connections would have entailed a high probability of receiving recurrent waves of pathogens^2^. Our simulation study fully supports this idea. We found that those cities having higher centrality and transitivity in the medieval network were infected more times by the plague than peripheral cities with low transitivity, irrespective of the plague infectivity level and the centrality metric used (Fig. 3). These theoretical findings agree with some empirical evidence also showing that re-infection was common in central cities. For example, multiple bacterial strains invaded London, a central city in our network, during the Black Death pandemic^31^.

When a city is recurrently invaded, the overall mortality will be higher due to several, non-exclusive reasons. A first factor increasing the mortality rate in a given city could be due to different infection waves affecting different parts of the city. In this case, the overall mortality rate will be the cumulative outcome of partial mortalities caused by each infection wave. In addition, a city invaded multiple times could receive different pathogen strains. Theoretical models suggest that multiple infections with different strains generally lead to the evolution of population-wide increased virulence^4^,^5^, magnifying even more the devastating effects of the epidemic in that city.

## Conclusions

Our findings may have important consequences for identifying epidemic hotspots. Our study suggests that the spread of the plague in Europe was related to the medieval trading and pilgrimage network. More importantly, our study provides quantitative support based on different network structural measures about a functional connection between the position of nodes (cities, villages, airports, harbors, etc.) in a network and their vulnerability to pandemics. We suggest that the mechanism behind this relationship is the higher recurrence of reinfection events in central nodes because the disease can reach them sooner and more often. It is indispensable to test how prevalent is this pattern nowadays^32^,^33^, where both the transportation network and the vulnerability of each node can be more accurately determined and where the topology of the entire network has probably changed as a consequence of many longer paths connecting more efficiently distant parts of the transportation networks. Knowing whether central nodes, besides fuelling the propagation of epidemics, are also more severely affected by diseases even in modern transportation networks is crucial to identify vulnerable human populations. This is specially relevant for those places in the world that are still isolated from the global transportation networks and where the spreading patterns may be pretty close to the patterns observed in medieval networks. If the history provides clues to understand the future, focusing our effort in vulnerable nodes may save time and resources by improving our ability of controlling deadly epidemics.

## Materials and Methods

To build the medieval trading and pilgrimage networks, we obtained the medieval routes from the Old World Trade Routes Project, OWTRAD^34^ (Supplementary Methods and Table S5). We checked whether the network followed a single-scale or a scale-free distribution by fitting the degree distribution to exponential and power-law functions using semi-log and log-log regressions, respectively. Afterwards, we calculated the global transitivity of each network and the local transitivity and centrality of each city. Local transitivity was calculated by local clustering coefficient^3^, whereas centrality was calculated by degree and closeness (Supplementary Methods). Degree is the number of nodes connected to a given node^3^. Closeness is the sum of the graph-distances from one node to all other nodes in the network^3^. The number of people dying to the plague was recorded using Benedictow^2^, Horrox^35^ and Sistach^36^ books and systematically searching in Google Scholar and SCOPUS including the words “Black Death”, “plague”, “mortality”, “survival”, and “death” (Supplementary Methods). When more than one value was found in a given city, we retained the most modern one or that having more support by historians (Supplementary Methods). To check the existence of spatial and temporal dynamics in mortality rates in our network, we obtained the spatial location of each city and the time of arrival of the plague to each city using the information provided by Büntgen *et al*.^37^ and Benedictow^2^ (Supplementary Methods). Spatio-temporal autocorrelation was checked by performing Mantel tests between the across-cities distances in mortality rate and the spatial and temporal distances (Supplementary Methods). The relationship between centrality and mortality rate during the Black Death pandemic was explored by fitting spatially-explicit generalised linear models. The dependent variable was mortality rate estimated as the proportion of the population dying due to the plague in each city. We performed five models, one including as independent variables the local transitivity values of the cities and the remaining models including each of the centrality metrics. To control for the time of arrival of the epidemics to the cities, we included this variable as covariate in all analyses. Finally, we simulated the diffusion of plague throughout our medieval network by using a susceptible-infectious-susceptible (SIS) epidemic model. This model describes how individuals change from susceptible to infected (Supplementary Methods). Individuals in our simulations were the cities, and the population was the entire set of cities connected through trading and pilgrimage routes (*N* = 1311 cities). In these models we allowed cities to be repeatedly infected without recovering from the diseases. Because our goal was to model how the plague moved among medieval cities following the trading and pilgrimage network, the transmission of the diseases was given by the contact network among cities (Supplementary Methods). The probability of a city of becoming infected depends thus on the number of infected cities contacting with the focal city multiplied by the transmission rate of the disease. Using a susceptible-infectious-susceptible (SIS) epidemic model, we simulated the spreading of the Black Death along the medieval network (Supplementary Methods, Appendix 3). We repeated the diffusion of the disease in several infectivity scenarios ranging between transmission rate of 0.05 (very low infectivity rate) to 0.95 (very high infectivity rate). We did 1000 simulations for each infectivity scenario. The epidemic was started in all simulations from central Asian cities. We obtained the number of times a given city was infected during each simulation (Appendix 3). Afterward, using the same statistical models explained in the previous section, we tested whether the number of infections was related to the centrality and transitivity of the cities.

## Additional Information

**How to cite this article**: Gómez, J. M. and Verdú, M. Network theory may explain the vulnerability of medieval human settlements to the Black Death pandemic. *Sci. Rep.* **7**, 43467; doi: 10.1038/srep43467 (2017).

**Publisher's note:** Springer Nature remains neutral with regard to jurisdictional claims in published maps and institutional affiliations.

## Supplementary Material

Supplementary Information

## Figures and Tables

**Table 1:** Spatially-explicit GLMs showing the effect of each centrality metric on mortality.

	Coefficient ± 1 SE	t	p-value
Overall network			
Local transitivity	33.82 ± 10.98	3.08	0.003
Degree centrality	4.60 ± 2.16	2.13	0.037
Closeness centrality	26.92 ± 12.90	2.09	0.042
Trade network			
Local transitivity	32.84 ± 10.59	3.10	0.003
Degree centrality	5.46 ± 2.78	1.96	0.054
Closeness centrality	1.13 ± 0.99	1.13	0.263
Pilgrimage network			
Local transitivity	Not applicable		
Degree centrality	1.19 ± 3.78	0.32	0.753
Closeness centrality	4.14 ± 5.50	0.75	0.456

All models include as a covariate the year of arrival of the plague to each city. It is shown the outcome from the complete network and from the networks built using only trading and pilgrimage links, respectively. Transitivity in the pilgrimage network could not be calculated because all the triples were open.
