# Supplementary material for: Network theory may explain the vulnerability of medieval human settlements to the Black Death pandemic
Source: Sci Rep. 2017 Mar 6;7:43467. doi: 10.1038/srep43467 (PMC5338018; doi:10.1038/srep43467)
Supplement: Supplementary Information [file srep43467-s1.pdf]

Supplementary Information

Network theory may explain the vulnerability of medieval human settlements to the Black Death pandemic

José M. Gómez and Miguel Verdú

Supplementary Methods

Building of the medieval trading and pilgrimage network. Several main commercial confederations were active during Late Middle Ages. One of the most active one was the Silk Road, a trading and cultural network spanning along the Asian continent and connecting the Far East and the Mediterranean Sea. In the southern part of Europe there were two trade routes connecting by sea and land Genoa and Venetia with many other cities in the Mediterranean, Northern Africa, Near East, Asia and central Europe. In northern Europe the Hanseatic League connected many ports around the Baltic Sea with cities from North and North-East Europe. Some other minor commercial routes like English Wool Market, Spanish Wine Market or North-African Slave trades were also present at that time. Besides these trading routes, Europe also held some important pilgrimage routes, outstanding the Way of St. James, which connected Santiago de Compostela in Spain with many places in Central and Western Europe, the Via Francigena, which run from Rome to France, although reached places as far as Canterbury in England and the North-African Islamic routes that connected muslim cities with La Mecca in the Arabian Peninsula.

To build the medieval trading and pilgrimage networks, we obtained the medieval routes from the Old World Trade Routes Project, OWTRAD[34]. This project provides electronic archives of geo/chrono-referenced data on terrestrial and maritime trade and pilgrimage and trade routes of Eurasia and Africa from 4000 BCE to 1800 CE. We selected those files describing the routes during the XII and XIII centuries CE. In total, we found 22 files with information about routes working during the Black Death period (Supplementary Table S5 online)

Using this information, we built up a network that connected Eurasian and African medieval cities sharing trading or pilgrimage routes. This network was built by pooling the information from the 22 files. Because the traffic may occurs in both directions, our network was undirected. Some pair of cities were connected both by trading and pilgrimage routes. However, because information on traffic density is unavailable for the fourteenth century, the original network was unweighted and all connections between pair of cities had the same value = 1. Nevertheless, in order to explore whether trading and pilgrimage activities differed in their effect on plague-mediated mortality, we divided this overall network into two networks, one including only trading links (trading network, hereafter) and the

other including only pilgrimage connections (pilgrimage network). For this, we followed the classification of the routes made by the OWTRAD project[34].

Global and local transitivity. Network transitivity measures the probability that adjacent nodes in a network are connected forming clusters (3). Networks exhibiting higher values of transitivity are formed by highly connected subsets of nodes. Network transitivity is calculated by dividing the number of closed triples in the graph against the total number of triples[3]. To know whether this transitivity was significant, we first made 100 random networks with the same size and density. In addition, we also made reshuffled networks with the same size and density as our empirical networks, maintaining the same degree of each of the nodes but reshuffling randomly the links among them. We did 100 reshuffling per network instance. In both cases we calculated the mean and 95% confidence interval of the transitivity values for this set of random networks[3].

Local transitivity measures the probability that neighboring cities of a focal city are also connected among them by direct routes. It was calculated by the local clustering coefficient, estimated as the ratio of the number of pairs of neighbors of a given node that are connected to the number of pairs of neighbors of that node[3]:

$$CC(i) = \frac{R(i)}{k(i)-1}$$

where R(*i*) is the mean number of connections from a neighbour of node *i* to other neighbors of node *i*, and k(*i*) is the degree of the node *i*[3]. These analyses were computed with the R packages igraph[38] and tnet[44].

Centrality Indices. The importance of a node within a network is quantified by its centrality[3, 18, 39]. Centrality indicates how well connected is a given city with the rest of the cities owing to shared routes. The centrality of the cities in the network was calculated by means of a local (degree) and a global (closeness) index[3, 18, 39, 40]. Each of these metrics captures different and complementary aspects of centrality.

Degree centrality can be defined as the number of links incident upon a node[40]. In epidemic networks, degree centrality appropriately measures short-term vulnerability of nodes to infection

because it indicates the proportion of times that the node is visited by the flow process in the network[22]. Degree centrality is the number of connected nodes to a given node, or number of adjacent nodes[3]. It can be formalised as:

$$k^w(i) = \sum_j^N w_{ij}$$

where w is the weighted adjacency matrix, in which w_{ij} is greater than 0 if the node i is connected to node j, and the value represents the weight of the edge. This is equal to the definition of degree if the network is binary, i.e. each edge has a weight of 1.

Closeness is the sum of the graph-distances from one node to all other nodes in the network[3]. Closeness defines the flow pathways, which has been shown to be important in infection spreading and can be interpreted as the expected time until arrival to a given node of something flowing through the network[12, 40]. It assesses the importance of a node based on its reachability within a network. Closeness centrality is the inverse sum of shortest distances to all other nodes from a focal node, and describes how close is a given node from the rest of the nodes in the network. Dijkstra's[41] algorithm finds the path of least resistance, defined for networks where weights represented costs of transmitting. Newman[42] and Brandes[43] inverted the edge weights to indicate that the weight of an edge between two nodes is directly proportional to the ease of flow between nodes. According to this idea, the distance between two nodes i and j is:

$$d^w(i,j) = \min\left(\frac{1}{w_{ih}} + \cdots + \frac{1}{w_{hj}}\right)$$

Using this computation of the distance between two given nodes, weighted closeness centrality is computed as:

$$C_C^w = \left[\sum_j^N d^w(i,j)\right]^{-1}$$

In general, degrees assess the importance of a node based on its reachability within a network. This measure provides a description of network connectivity based on the individual components. Closeness

defines the flow pathways, which has been shown to be important in infection spreading. Nodes with high values of these metric act as bridges, connecting one part of a network to another that would otherwise be sparsely or not connected at all, favouring the spreading of disease across the entire network[3].

Centrality indices were computed with the R packages igraph[38] and tnet[44].

Calculation of the mortality rate due to the plague. The number of people dying to the plague was recorded using Benedictow[2], Horrox[35] and Sistach[36]. In addition, we systematically searched for extra information on mortality. For this, we carried out computer searches in Google Scholar and SCOPUS including the words "Black Death", "plague", "mortality", "survival", and "death". We only retrieved the information referring to the Black Death epidemics. When more than one value was found in a given city, we retained the most modern one or that having more support by historians.

Calculation of the spatio-temporal correlation in mortality rate. To check the existence of spatial and temporal dynamics in mortality rates in our network, we obtained the spatial location of each city and the time of arrival of the plague to each city using the information provided by Büntgen et al.[37] and Benedictow[2].

Spatio-temporal autocorrelation was checked by performing Mantel tests between the across-cities distances in mortality rate and the spatial and temporal distances. In these analyses, we used the Euclidean distance in mortality rates. The temporal distance matrix was calculated as the difference among cities in the year of the plague's arrival. Geographic distances can be calculated using several measures. Travelling distances can be a realistic estimate of the distance between two cities but, unfortunately, there is not accurate information about these measures in the medieval network and therefore we used geodesic distance between cities as a proxy. The Mantel tests were performed with the R package vegan[45].

Spatially-explicit generalised linear models. The relationship between centrality and mortality rate during the Black Death pandemic was explored by fitting spatially-explicit generalised linear models. The dependent variable was mortality rate estimated as the proportion of the population dying due to the

plague in each city. We performed five models, one including as independent variables the local transitivity values of the cities, and the remaining models including each of the centrality metrics (degree and closeness), respectively. We repeated these analyses for the three networks considered here, the overall network, the trade network and the pilgrimage network. To control for the time of arrival of the epidemics to the cities, we included this variable as covariate in all analyses. All explanatory variables except time of arrival were log-transformed.

In addition, we generated random networks with the same size (number of nodes) and number of links than the three studied networks (overall, trade and pilgrimage) using the Erdos-Renyi G(n,m) model. According to this model, the random network is chosen uniformly at random from a set of network having n nodes and m links. We then calculated for each city their centrality and transitivity in these random networks. Using the spatially-explicit generalised linear models, we checked whether the relationship between the network attributes of the cities and the mortality rate due to the plague maintain in these random networks.

All the statistical analyses were performed using the R packages igraph[38] and nlme[46].

Simulating the effect of centrality and transitivity on the probability of multiple infections. We simulated the diffusion of plague throughout our medieval network by using a susceptible-infectious-susceptible (SIS) epidemic model. This model describes how individuals change from susceptible to infected. This model is described by two standard sets of differential equations:

$$\frac{dS}{dt} = gI - \lambda S$$

$$\frac{dI}{dt} = \lambda S - gI$$

where S and I refer to the number of susceptible and infectious individuals, respectively, in a population of size *N*, g is the rate of recovery from infection, and λ is the rate at which susceptible individuals become infected. Individuals in our simulations were the cities, and the population was the entire set of cities connected through trading and pilgrimage routes (*N* = 1311 cities). Because a city can be repeatedly infected without recovering from the diseases, we simplified the above equations by considering g = 0. We did this simplification because a city usually contains both infected and susceptible individuals. If this occurs, the city is considered to be infected although some individuals are

susceptible. Under a scenario of re-infection, susceptible individuals can be infected before the recovery of the other individuals in the city. Nevertheless, it would be convenient in the future to test the response of the model to more complex scenarios, like for example, modelling the proportion of the population that is infected within cities.

According to this model, every individual from the population will be eventually infected. In addition, in a closed population without birth, death and migration, and mixing at random, all individuals have the same probability of becoming infected. Under these circumstances, the rate of infection can be calculated as:

$$\lambda = \beta \times \hat{n} \times \frac{I}{N}$$

where β is the transmission rate (the probability of the disease to infect a susceptible city when contacting with an infected city), $\hat{n}$ is the effective number of contacts per unit time and *I/N* is the proportion of infected contacts. The SIS models, by considering random mixing, assume that each city has a small and equal chance of contacting with any other city in the population. So, $\hat{n}$ is equal to 1, and this equation becomes:

$$\lambda = \beta \times \frac{I}{N}$$

Our goal was to model how the plague moved among medieval cities following the trading and pilgrimage network. This means that the assumption of random mixing is not fulfilled. Rather, the transmission of the diseases is given by the contact network among cities. The probability of a city of becoming infected depends thus on the number of infected cities contacting with the focal city multiplied by the transmission rate of the disease. We included this constraint in our modelling approach by considering as contact network the empirical medieval network depicting trade and pilgrimage routes.

Using SI epidemic models, we simulated the spreading of the Black Death along the medieval network. Because no accurate information exists on the transmission rate of the Black Death, we repeated the diffusion of the disease in several infectivity scenarios ranging between transmission rate of 0.05 (very low infectivity rate) to 0.95 (very high infectivity rate). We did 1000 simulations for each infectivity scenario. The epidemic was started in all simulations from central Asian cities. We obtained the number of times a given city was infected during each simulation. Afterward, using the same

statistical models explained in the previous section, we tested whether the number of infections was related to the centrality and transitivity of the cities. The script used to make the simulations is provided in SI Dataset.

References

37. Büntgen, U., Ginzler, C., Esper, J., Tegel, W. & McMichael, A. J. Digitizing historical plague. *Clin. Infect. Dis*. **55**, 1586–1588 (2012).
38. Csardi, G. & Nepusz, T. The igraph software package for complex network research. *Int. J. Complex Syst.* 1695 (2006).
39. Opsahl, T., Agneessens, F. & Skvoretz, J. Node centrality in weighted networks: generalizing degree and shortest paths. *Soc. Networks* **32**, 245-251 (2010).
40. Borgatti, S. P. Centrality and network flow. *Soc. Networks* **27**, 55-71 (2005).
41. Dijkstra, E. W. A note on two problems in connexion with graphs. *Numerische Mathematik* **1**, 269–271 (1959).
42. Newman, M. E. J. Scientific collaboration networks. II. Shortest paths, weighted networks, and centrality. *Phy. Rev.* **E64**, 016132 (2001).
43. Brandes, U. A faster algorithm for betweenness centrality*. J. Math. Sociol.* **25**, 163–217 (2001).
44. Opsahl, T. *Structure and Evolution of Weighted Networks* (University of London, Queen Mary College, 2009).
45. Oksanen, J., Blanchet, F. G., Kindt, R., Legendre, P., Minchin, P. R., et al. *vegan: community ecology package*. R package version 2.2, (2013).
46. Pinheiro, J., Bates, D., DebRoy, S., Sarkar, D. & R Core Team. *nlme: Linear and Nonlinear Mixed Effects Models*. R package version 3.1-121 (2015).


```
################################################################################
############             Drawing a spatial network of European trade            ############
################################################################################
require(sp); require (maps); require(maptools); require(mapdata); require (scales); require
  (igraph); require (tnet)

cities<-read.table ("Data.OWTRAD.cities.txt", header =T, row.names=1)
```

```
OWTRAD=read.csv("Data.OWTRAD.Edgelist.txt", header =TRUE)
OWTRAD =as.matrix(OWTRAD) #igraph needs the edgelist to be in matrix format
g=graph.edgelist(OWTRAD[,1:2], directed=FALSE)
E(g)$weight=as.numeric(OWTRAD[,3])
E(g)$type.of.route=as.factor(OWTRAD[,4])

#### Ordering g and spatial.city to assign right spatial coordinates

City<-V(g)$name
City<-data.frame(City)
cities.ordered<-cities[City$City,]

#cities.ordered$Arrival<-as.factor(cities.ordered$Arrival)

# to associate spatial coordinates as vertex attributes
V(g)$lat<-cities.ordered$y
V(g)$lon<-cities.ordered$x
gV <- SpatialPoints(cbind(V(g)$lon, V(g)$lat))
#writePointsShape(gV, fn="vertices")


# Create the SpatialLinesDataFrame object for the edges.
#Needs numeric edgelist in Pajek format
#write.graph(g, file="Data.OWTRAD.Edgelist.num.txt", format="pajek")
OWTRAD.num=read.csv("Data.OWTRAD.Edgelist.num.txt", header =TRUE)
OWTRAD.num =as.matrix(OWTRAD.num) #igraph needs the edgelist to be in matrix format
g.num=graph.edgelist(OWTRAD.num[,1:2], directed=FALSE)
E(g.num)$weight=as.numeric(OWTRAD.num[,3])
#E(g.num)$trade=as.factor(OWTRAD.num[,4])
edges <- get.edgelist(g.num)
edges <- cbind(edgeNum=1:nrow(edges), city1=edges[,1], city2=edges[,2])

## Create SpatialLinesDataFrame object describing edges
gE <- apply(edges, 1, function(i) Lines(Line(cbind(c(V(g)$lon[i["city1"]], V(g)$lon[i["city2"]]),
 c(V(g)$lat[i["city1"]], V(g)$lat[i["city2"]]))), ID=as.character(i["edgeNum"])))
gE <- SpatialLinesDataFrame(SpatialLines(gE), data=data.frame(edgeNum=1:nrow(edges)))

## Write edges to a shapefile
#writeLinesShape(gE, fn="edges")


#Drawing the spatially-explicit network
map (xlim=c(-30, 150), ylim=c(0, 70), col="gray90", fill=TRUE)
box()
map.axes()
map.scale(x=-29, y=2, ratio=FALSE, relwidth=0.2)
lines(gE, col ="blue")
points(gV, pch=21, cex=1+cities.ordered$degree/3, xlab="longitude", ylab="latitude", col=
 "black", bg=111)
points(gV, pch=21, cex=1+cities.ordered$Prin1/2, xlab="longitude", ylab="latitude", col=
 "black", bg= c("gray", "blue", "red", "yellow", "darkviolet", "green", "magenta",
 "black")[cities.ordered$Arrival])
```

```
####### Drawing different routes with different colours
# Drawing the trade network [type value= 1]

OWTRAD.num=read.csv("Data.OWTRAD.Edgelist.num.txt", header =TRUE) # read the file
OWTRAD.num.trade=OWTRAD.num[OWTRAD.num$type!=2 & OWTRAD.num$type!=3, ]
OWTRAD.num.trade=as.matrix(OWTRAD.num.trade) #igraph needs the edgelist to be in matrix
 format
g.num.trade=graph.edgelist(OWTRAD.num.trade[,1:2], directed=FALSE)
E(g.num.trade)$weight=as.numeric(OWTRAD.num.trade[,3])
E(g.num.trade)$trade=as.factor(OWTRAD.num.trade[,4])
edges.trade <- get.edgelist(g.num.trade)
edges.trade <- cbind(edgeNum=1:nrow(edges.trade), city1=edges.trade[,1],
 city2=edges.trade[,2])
gE.trade <- apply(edges.trade, 1, function(i) Lines(Line(cbind(c(V(g)$lon[i["city1"]],
 V(g)$lon[i["city2"]]), c(V(g)$lat[i["city1"]], V(g)$lat[i["city2"]]))),
 ID=as.character(i["edgeNum"])))
gE.trade <- SpatialLinesDataFrame(SpatialLines(gE.trade),
 data=data.frame(edgeNum=1:nrow(edges.trade)))

writeLinesShape(gE.trade, fn="edges")

# Drawing the pilgrim network [type value= 2]

OWTRAD.num=read.csv("Data.OWTRAD.Edgelist.num.txt", header =TRUE) # read the file
OWTRAD.num.pilgrim=OWTRAD.num[OWTRAD.num$type!=1 & OWTRAD.num$type!= 3, ]
OWTRAD.num.pilgrim=as.matrix(OWTRAD.num.pilgrim) #igraph needs the edgelist to be in
 matrix format
g.num.pilgrim=graph.edgelist(OWTRAD.num.pilgrim[,1:2], directed=FALSE)
E(g.num.pilgrim)$weight=as.numeric(OWTRAD.num.pilgrim[,3])
E(g.num.pilgrim)$trade=as.factor(OWTRAD.num.pilgrim[,4])
edges.pilgrim <- get.edgelist(g.num.pilgrim)
edges.pilgrim <- cbind(edgeNum=1:nrow(edges.pilgrim), city1=edges.pilgrim[,1],
 city2=edges.pilgrim[,2])
gE.pilgrim <- apply(edges.pilgrim, 1, function(i) Lines(Line(cbind(c(V(g)$lon[i["city1"]],
 V(g)$lon[i["city2"]]), c(V(g)$lat[i["city1"]], V(g)$lat[i["city2"]]))),
 ID=as.character(i["edgeNum"])))
gE.pilgrim <- SpatialLinesDataFrame(SpatialLines(gE.pilgrim),
 data=data.frame(edgeNum=1:nrow(edges.pilgrim)))

writeLinesShape(gE.pilgrim, fn="edges")

# Drawing the courier network [trade value= 3]

OWTRAD.num=read.csv("Data.OWTRAD.Edgelist.num.txt", header =TRUE) # read the file
OWTRAD.num.courier=OWTRAD.num[OWTRAD.num$type!=1 & OWTRAD.num$type!= 2, ]
OWTRAD.num.courier=as.matrix(OWTRAD.num.courier) #igraph needs the edgelist to be in
 matrix format
g.num.courier=graph.edgelist(OWTRAD.num.courier[,1:2], directed=FALSE)
E(g.num.courier)$weight=as.numeric(OWTRAD.num.courier[,3])
E(g.num.courier)$trade=as.factor(OWTRAD.num.courier[,4])
edges.courier <- get.edgelist(g.num.courier)
```

```
edges.courier <- cbind(edgeNum=1:nrow(edges.courier), city1=edges.courier[,1],
 city2=edges.courier[,2])
gE.courier <- apply(edges.courier, 1, function(i) Lines(Line(cbind(c(V(g)$lon[i["city1"]],
 V(g)$lon[i["city2"]]), c(V(g)$lat[i["city1"]], V(g)$lat[i["city2"]]))),
 ID=as.character(i["edgeNum"])))
gE.courier <- SpatialLinesDataFrame(SpatialLines(gE.courier),
 data=data.frame(edgeNum=1:nrow(edges.courier)))

writeLinesShape(gE.courier, fn="edges")

map (xlim=c(-20, 130), ylim=c(0, 65), col="grey", fill=TRUE)

#box()
#map.axes()
map.scale(x=-29, y=2, ratio=FALSE, relwidth=0.2)
lines(gE.trade, col ="blue")
lines(gE.pilgrim, col ="white")
lines(gE.courier, col ="blue")
points(gV, pch=21, cex=1+cities.ordered$degree/8, xlab="longitude", ylab="latitude", col=
 "blue", bg="blue")


##############################################################################
############     Modelling diffusion process in medieval networks in R          ############
##############################################################################
require(networkdiffusion); require(igraph); require(network); require(nlme)

# Generate a contact network using the medieval network
OWTRAD=read.csv("Data.OWTRAD.Edgelist.noduplicados.txt", header =TRUE) # read the file
OWTRAD=as.matrix(OWTRAD) #igraph needs the edgelist to be in matrix format
g=graph.edgelist(OWTRAD[,0:2], directed=FALSE)
E(g)$weight=as.numeric(OWTRAD[,4])

# Loading city datasets with centrality and transitivity values
cities<-read.table ("Data.OWTRAD.cities.2.txt", sep=",", header=TRUE) # We used the small
 dataset with few columns

# Model parameters
transmission.rate = c(0.05, 0.15, 0.25, 0.35, 0.45, 0.55, 0.65, 0.75, 0.85, 0.95) # M
starting.nodes=c(477,547,676,677,678,679,680,681,682,689,742,766,767,769,770,807,808,81
 3,814,909,965,987,988,989,1009,1023,1028,1029,1034,1075,1093,1105,1161,1167,1206,120
 7,1234,1235,1278) # The code number of Asian cities
#starting.nodes=c(1:1311)

seed=NULL

for(i in 1:1000){
seed[i]= sample(starting.nodes,1) # Sampling seeds from Asian cities
diffusers = V(g)[seed[i]]
infected =list()
infected[[1]]= diffusers
```

```
# Set percolation probability
coins = c(1, 0)
probabilities = c(transmission.rate, 1-transmission.rate )
# toss the coins
toss = function(freq) {
  tossing = NULL
  for (i in 1:freq ) tossing[i] = sample(coins, 1, rep=TRUE, prob=probabilities)
  tossing = sum(tossing)
  return (tossing)
                    }

# Allowing multiple infections
 update_diffusers = function(diffusers){
   nearest_neighbors = data.frame(table(unlist(neighborhood(g, 1, diffusers))))
   keep= unlist(lapply(nearest_neighbors[,2], toss))
   new_infected = as.numeric(as.character(nearest_neighbors[,1][keep>=1]))
   diffusers = c(diffusers, new_infected)
   return(diffusers)
                    }
# Start the contagion, allowing the disease to infect 6 times the number of nodes in the
 contact network to ensure multiple infection
total_time = 1
while(length(infected[[total_time]]) < (6*node_number)){
  infected[[total_time+1]] = sort(update_diffusers(infected[[total_time]]))
  total_time = total_time + 1
                    }

# Outcomes files- Files with multiple infections
infected[length(infected)]-> final.infected
final.infected <-as.data.frame(final.infected)
names(final.infected)<-paste("nodes")
final.infected$no_infections<-1
multiple.infection<-aggregate(final.infected$no_infections, by=
 list(nodes=final.infected$nodes), FUN=sum)
names(multiple.infection)[2]<-paste("no_infections")

# Outcome files- Files with city names
v<-get.data.frame(g, what="vertices")
nodes<-1:1311
v$nodes<-nodes
names(v)[1]<-paste("city")

temporal.file<-merge(v, multiple.infection, by="nodes", all=TRUE)
temporal.file[is.na(temporal.file)]<-0

merge(cities, temporal.file, by="city", all=TRUE)->vb

# Testing the effect of centrality and transitivity on re-infection
gauss.mod.degree<-gls(no_infections~log(degree), data= vb)
gauss.mod.degree<-update(mod.degree,corr=corGaus(form=~x+y))

gauss.mod.closeness<-gls(no_infections ~log(closeness+1), data= vb)
```

```
gauss.mod.closeness<-update(mod.closeness,corr=corGaus(form=~x+y))


gauss.mod.transitivity<-gls(no_infections ~log(transitivity.overall+1), na.action=na.omit, data=
 vb)
gauss.mod.transitivity<-update(mod.transitivity,corr=corGaus(form=~x+y))

                  }
```

FIGURES

Figure S1. Degree distribution. It is shown the cumulative distribution of number of links per node (*k*, degree).

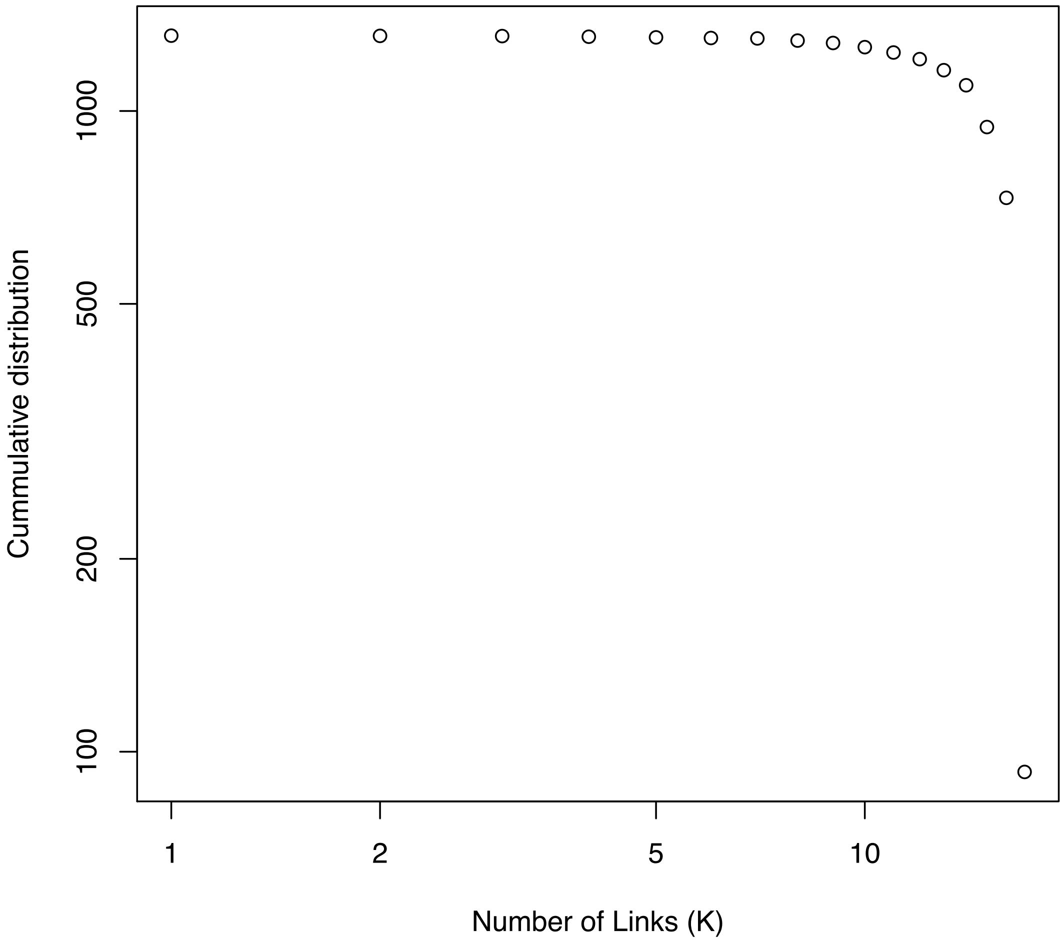

Figure S2. Box plot showing the median, the 25%, 75% and 95% values of the clustering coefficients in the overall network as well as in the trade and pilgrimage networks.

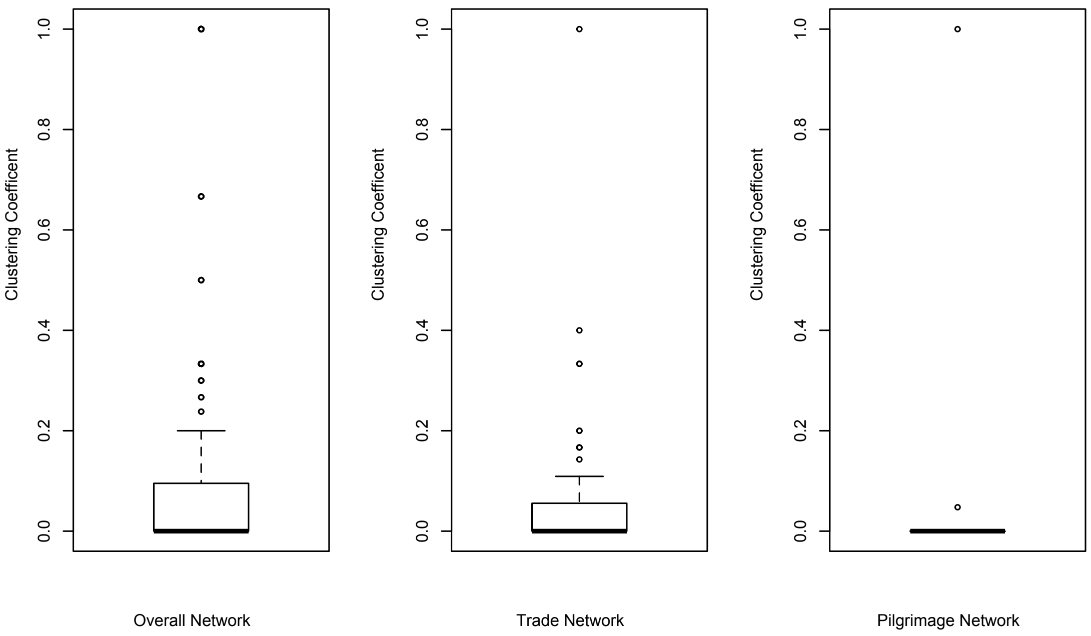

Figure S3. Box plot showing the median, the 25%, 75% and 95% values of the two indices of centrality in the overall network.

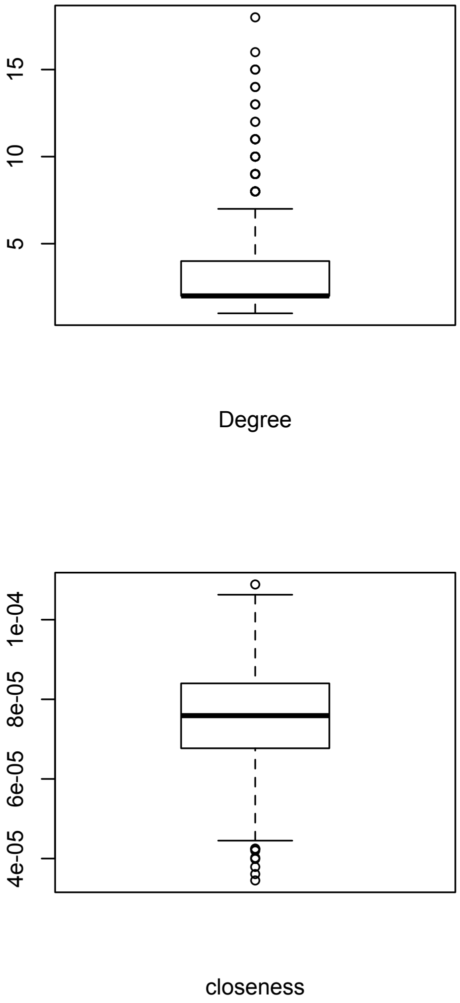

Figure S4. Box plot showing the median, the 25%, 75% and 95% values of the two indices of centrality in the trade network.

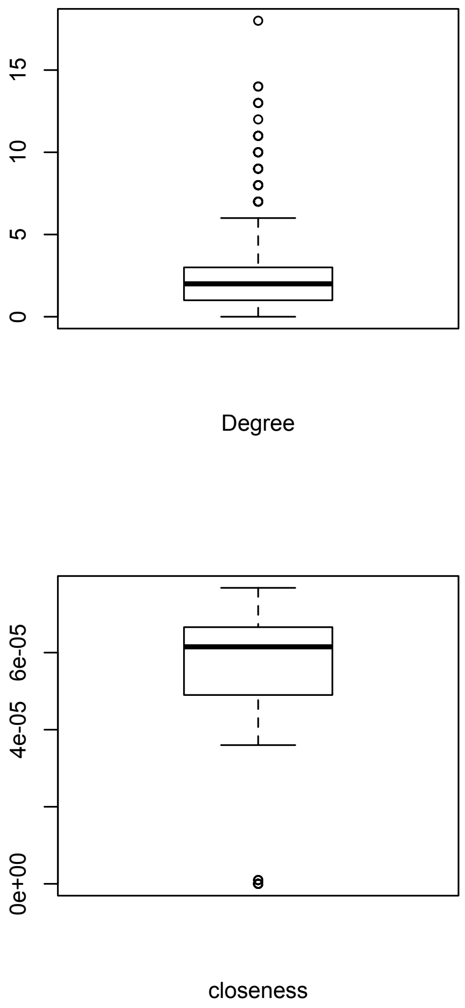

Figure S5. Box plot showing the median, the 25%, 75% and 95% values of the two indices of centrality in the pilgrimage network.

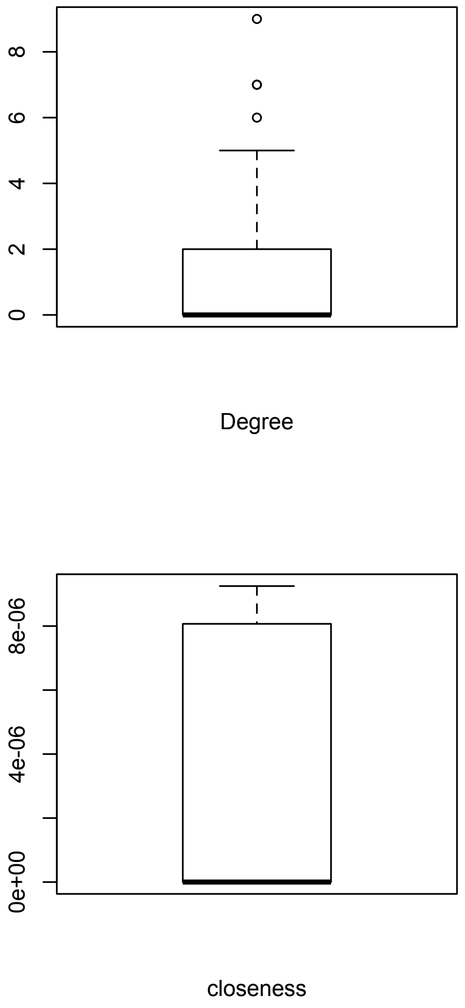

Figure S6. Relationship between the clustering coefficient and the degree (grouping all nodes with same degree) for both trade and overall networks. (pilmigrame network had too few non-zero clustering coefficient values).

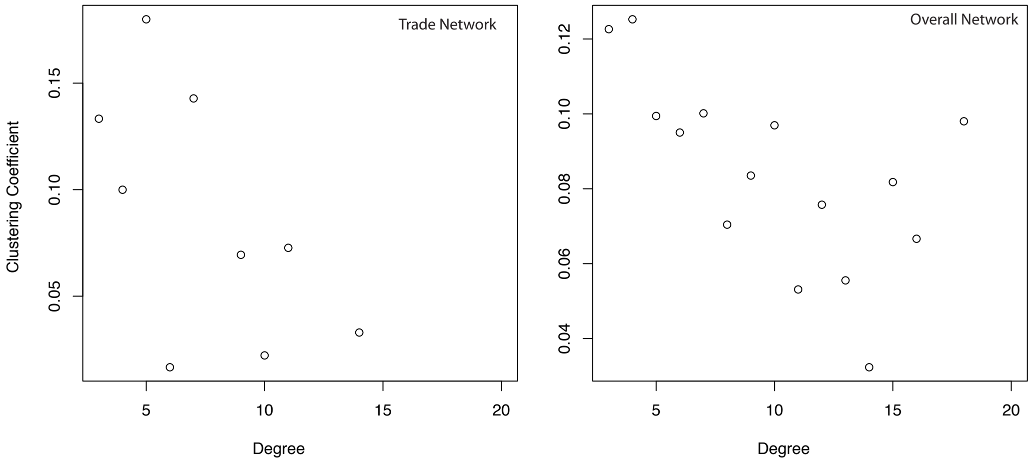

Table S1. Summary of the centrality metrics used in this study in each of the three types of networks. Reshuffled network transitivity were obtained for 100 networks maintaining the same degree distribution across nodes but reshuffling randomly the links. Random network transitivity were obtaining for 100 networks under a Erdos-Rényi G(n,m) model and with same size and density than the observed networks.

	Overall network	Trading network	Pilgrimage Network
Number of routes	2084	1634	451
Number of cities having routes	1311	1013	403
Network density	0.0024	0.0032	0.0056
Network transitivity	0.1053	0.1135	0.0530
Reshuffled network transitivity	0.001 (0.000-0.003)	0.006 (0.002-0.012)	0.000 (0.000-0.000)
Random network transitivity	0.0024 (0.0022-0.0036)	0.0032 (0.0030-0.0034)	0.0060 (0.0051-0.0068)

Table S2. Outcome of the Pearson correlations between degree and closeness for each type of network (overall, trade and pilgrimage networks). It is also shown the mean and the 95 % confidence interval of the correlations in null networks made by reshuffling the links randomly across nodes but maintaining their degree.

Variable 1	r	P-value	95% CI of the Null Networks
Overall network	0.4494	<0.0001	0.4152 – 0.4222
Trade Network	0.6414	<0.0001	0.4099 – 0.4174
Pilgrimage Network	0.8920	<0.0001	0.3703 – 0.3871

Table S3. Outcome of the Pearson correlation between closeness centrality and the local clustering coefficient for each type of network (overall, trade and pilgrimage networks). It is also shown the mean and the 95 % confidence interval of the correlations in null networks made by reshuffling the links randomly across nodes but maintaining their degree.

Variable	r	P-value	95% CI of the Null Networks
Overall network	0.1866	<.0001	0.0063 – 0.0215
Trade network	0.0597	0.6560	0.0000 – 0.0131
Pilgrimage network	-0.0362	0.7871	0.0000 – 0.0410

Table S4. Main characteristics of medieval cities used in the analysis testing the relationship between network attributes and plague severity.

	Spatial location		overall network		trading Network		pilgrimage network			
City	long	lat	degree	closen.	degree	closen.	degree	closen.	Mortality	Arrival
Aix	5.260	43.310	2	0.0000821	2	0.0000640	0	0.0000000	45.0	1347
Ajaccio	8.440	41.550	3	0.0000951	3	0.0000698	0	0.0000000	75.0	1347
Arras	2.783	50.283	2	0.0000611	1	0.0000535	1	0.0000062	40.0	1349
Avignon	4.817	43.950	7	0.0000889	6	0.0000672	1	0.0000081	72.0	1348
Barcelona	2.183	41.383	10	0.0000801	10	0.0000625	0	0.0000000	74.0	1348
Beziers	3.130	43.200	2	0.0000745	2	0.0000598	0	0.0000000	72.0	1348
Bologna	11.333	44.483	8	0.0000980	5	0.0000723	3	0.0000092	45.0	1348
Bremen	8.800	53.083	6	0.0000765	5	0.0000618	1	0.0000070	60.0	1350
Bristol	-2.583	51.450	1	0.0000546	0	0.0000000	1	0.0000081	44.5	1348
Cagliari	9.117	39.217	1	0.0000709	1	0.0000585	0	0.0000000	50.0	1347
Cairo	31.250	30.050	5	0.0000769	3	0.0000622	2	0.0000083	57.1	1348
Calais	1.510	50.570	2	0.0000670	2	0.0000569	0	0.0000000	60.0	1348
Constantinople	28.967	41.017	14	0.0001008	14	0.0000761	0	0.0000000	50.0	1347
Cornwall	-4.090	50.300	1	0.0000590	1	0.0000516	0	0.0000000	21.0	1349
Dublin	-6.150	53.200	1	0.0000725	1	0.0000588	0	0.0000000	56.0	1348
Dubrovnik	18.089	42.651	9	0.0000906	9	0.0000692	0	0.0000000	50.0	1350
Durham	-1.567	54.767	1	0.0000669	1	0.0000569	0	0.0000000	50.0	1349
Erfurt	11.033	50.983	9	0.0000912	5	0.0000688	4	0.0000070	57.0	1350
Estella	-2.033	42.667	4	0.0000618	0	0.0000000	4	0.0000081	63.0	1348
Exeter	3.320	50.430	1	0.0000635	1	0.0000546	0	0.0000000	51.5	1349
Florence	11.250	43.767	6	0.0000892	4	0.0000681	2	0.0000092	65.0	1348
Genoa	8.950	44.417	7	0.0000985	7	0.0000713	0	0.0000000	52.0	1348
Gy	5.340	47.270	2	0.0000590	2	0.0000010	0	0.0000000	41.3	1348
Hamburg	10.000	53.550	5	0.0000765	5	0.0000619	0	0.0000000	67.0	1349

King’s_Lynn	0.400	52.750	1	0.0000489	1	0.0000458	0	0.0000000	57.0	1349
Lincoln	-0.540	53.230	1	0.0000734	1	0.0000604	0	0.0000000	40.2	1349
London	-0.117	51.500	10	0.0000811	10	0.0000643	0	0.0000000	57.0	1348
Magdeburg	11.667	52.167	8	0.0000824	6	0.0000648	2	0.0000070	50.0	1350
Marseilles	5.400	43.300	5	0.0000811	5	0.0000633	0	0.0000000	60.0	1348
Milan	9.200	45.467	13	0.0001039	11	0.0000737	2	0.0000092	10.0	1348
Monthey	5.617	46.150	2	0.0000795	2	0.0000634	0	0.0000000	52.5	1348
Montpellier	3.883	43.600	4	0.0000824	4	0.0000639	0	0.0000000	72.0	1348
Montreux	6.550	46.260	1	0.0000548	1	0.0000010	0	0.0000000	52.5	1349
Narbonne	3.000	43.183	6	0.0000795	6	0.0000622	0	0.0000000	58.0	1348
Newcastle	-1.370	54.580	1	0.0000669	1	0.0000569	0	0.0000000	30.0	1350
Norwich	1.170	52.370	1	0.0000734	1	0.0000604	0	0.0000000	48.8	1348
Padua	11.883	45.417	3	0.0000990	3	0.0000727	0	0.0000000	67.0	1348
Palma	2.650	39.567	6	0.0000782	6	0.0000622	0	0.0000000	23.0	1347
Pamplona	-1.633	42.817	4	0.0000681	2	0.0000549	2	0.0000081	65.0	1348
Paris	2.333	48.867	10	0.0000924	9	0.0000696	1	0.0000081	70.0	1349
Perpignan	2.883	42.683	5	0.0000777	4	0.0000614	0	0.0000000	67.0	1348
Pisa	10.383	43.717	4	0.0000894	4	0.0000678	0	0.0000000	50.0	1348
Poggibonsi San Gimignano	11.150	43.467	2	0.0000740	0	0.0000000	2	0.0000092	56.0	1348
Porcari	10.370	43.500	1	0.0000757	1	0.0000615	0	0.0000000	45.0	1348
San Giorgio	25.590	43.550	1	0.0000890	2	0.0000565	0	0.0000000	45.7	1348
Santiago de Compostela	-8.550	42.883	7	0.0000582	0	0.0000000	7	0.0000081	30.0	1348
Shrewsbury	-2.750	52.710	1	0.0000734	1	0.0000604	0	0.0000000	22.0	1349
Siena	11.350	43.317	4	0.0000819	2	0.0000646	2	0.0000092	60.0	1348
Skanor	12.510	55.250	1	0.0000682	1	0.0000576	0	0.0000000	50.0	1350
Stockholm	18.040	59.190	3	0.0000572	3	0.0000519	0	0.0000000	46.0	1350
Teruel	-10.060	40.200	1	0.0000725	1	0.0000588	0	0.0000000	37.0	1348
Turku	22.250	60.500	1	0.0000571	1	0.0000518	0	0.0000000	0.0	1351

Venice	12.327	45.439	13	0.0001088	11	0.0000768	2	0.0000092	67.0	1348
Vic	2.150	41.560	2	0.0000856	2	0.0000654	0	0.0000000	66.0	1348
Visby	18.300	57.633	4	0.0000618	4	0.0000546	0	0.0000000	59.0	1350
Winchester	-1.310	51.060	1	0.0000734	1	0.0000604	0	0.0000000	48.8	1348
Worcester	-2.200	52.200	1	0.0000669	1	0.0000569	0	0.0000000	44.5	1349
York	-1.083	53.967	3	0.0000734	3	0.0000604	0	0.0000000	44.2	1349

Table S5. List of files used in the network analyses. All files were obtained from the Old World Trade Routes Project -OWTRAD- webpage (http://www.ciolek.com/owtrad.html).

**Files used in the network analyses**
Old World Trade Routes (OWTRAD)- The Silk Road from the Adriatic to the Pacific 1200-1400 CE
Old World Trade Routes (OWTRAD)- Chief trade routes in Europe, Levant and North Africa 1300-1500 CE
Old World Trade Routes (OWTRAD)- North African pilgrimage routes 1300-1900 CE
Old World Trade Routes (OWTRAD)- Main trade routes in the Holy Roman Empire and nearby countries, c. 1500 CE
Old World Trade Routes (OWTRAD)- Courier routes connecting banking places in Western Europe 1370-1430 CE
Old World Trade Routes (OWTRAD)- Venetian galley-operated trade routes 1400-1530 CE
Old World Trade Routes (OWTRAD)- Trade routes in SE Poland and Ukraine 1200-1700 CE
Old World Trade Routes (OWTRAD)- Major trade roads in Poland and adjacent border regions 1200-1450 CE
Old World Trade Routes (OWTRAD)- Major trade roads in Poland and adjacent border regions in 1370 CE
Old World Trade Routes (OWTRAD)- Major major roads in Poland and adjacent regions c. 1150 CE
Old World Trade Routes (OWTRAD)- South German trade routes before 1500 CE
Old World Trade Routes (OWTRAD)- Central European pilgrimage routes to Rome c. 1500 CE
Old World Trade Routes (OWTRAD)- Woollen cloth trade routes in North-Western Europe 1100-1500 CE
Old World Trade Routes (OWTRAD)- Spanish pilgrimage routes 900-2000 CE
Old World Trade Routes (OWTRAD)- NW African trade routes 500-1900 CE
Old World Trade Routes (OWTRAD)- Moroccan and Trans-Saharan trade routes 200-1930 CE
Old World Trade Routes (OWTRAD)- Silk Road routes 1-1400 CE
Old World Trade Routes (OWTRAD)- Trade routes in the Ottoman Empire 1300-1600 CE
Old World Trade Routes (OWTRAD)- The Anatolian Silk Road 1200-1400 CE
Old World Trade Routes (OWTRAD)- Islamic trade and pilgrimage routes 1300-1600 CE
Old World Trade Routes (OWTRAD)- Silk Road routes between the Mediterranean, Iran and China 200 BCE-1400 CE
Old World Trade Routes (OWTRAD)- French pilgrimage routes 1000-1500 CE.
